# Diagnosis, management and post-mortem findings of a human case of rabies imported into the United Kingdom from India: a case report

**DOI:** 10.1186/1743-422X-11-63

**Published:** 2014-04-07

**Authors:** Smriti Pathak, Daniel L Horton, Sebastian Lucas, David Brown, Shumonta Quaderi, Sara Polhill, David Walker, Eleni Nastouli, Alejandro Núñez, Emma L Wise, Anthony R Fooks, Michael Brown

**Affiliations:** 1 Hospital for Tropical Diseases, Virology and Intensive Care Units, University College Hospitals NHS Foundation Trust, London, UK; 2 Animal Health and Veterinary Laboratories Agency (AHVLA), Wildlife Zoonoses and Vector-borne Diseases Research Group, Department of Virology, Weybridge, Surrey KT15 3NB, UK; 3 Public Health England (formally Health Protection Agency), London, UK; 4 Department of Clinical Infection, Microbiology and Immunology, University of Liverpool, Liverpool L3 5TQ, UK; 5 London School of Hygiene and Tropical Medicine, London, UK; 6 Department of Histopathology, St Thomas’ Hospital, London, UK

**Keywords:** Rabies, Milwaukee protocol, Diagnosis

## Abstract

**Background:**

Human rabies infection continues to be a significant public health burden globally, and is occasionally imported to high income settings where the Milwaukee Protocol for intensive care management has recently been employed, with limited success in improving survival. Access to molecular diagnostics, pre- and post-mortem, and documentation of pathophysiological responses while using the Milwaukee protocol, can add useful insights for the future of rabies management.

**Case presentation:**

A 58-year-old British Asian woman was referred to a regional general hospital in the UK with hydrophobia, anxiety and confusion nine weeks after receiving a dog bite in North West India. Nuchal skin biopsy, saliva, and a skin biopsy from the site of the dog bite wound, taken on the day of admission, all demonstrated the presence of rabies virus RNA. Within 48 hours sequence analysis of viral RNA confirmed the diagnosis and demonstrated that the virus was a strain closely related to canine rabies viruses circulating in South Asia. Her condition deteriorated rapidly with increased agitation and autonomic dysfunction. She was heavily sedated and intubated on the day after admission, treated according to a modified Milwaukee protocol, and remained stable until she developed heart block and profound acidosis and died on the eighth day. Analysis of autopsy samples showed a complete absence of rabies neutralizing antibody in cerebrospinal fluid and serum, and corresponding high levels of virus antigen and nucleic acid in brain and cerebrospinal fluid. Quantitative PCR showed virus was also distributed widely in peripheral tissues despite mild or undetectable histopathological changes. Vagus nerve branches in the heart showed neuritis, a probable Negri body but no demonstrable rabies antigen.

**Conclusion:**

Rapid molecular diagnosis and strain typing is helpful in the management of human rabies infection. Post-mortem findings such as vagal neuritis highlight clinically important effects on the cardiovascular system which are typical for the clinical course of rabies in humans. Management guided by the Milwaukee protocol is feasible within well-resourced intensive care units, but its role in improving outcome for canine-derived rabies remains theoretical.

## Background

Rabies continues to cause an estimated 24,000 -93,000 human deaths worldwide [1]. With an over-representation of children among these cases, an estimated 1.74 (95% CI 0.75-2.93) million disability adjusted life years are lost annually [1,2]. Rabies persists in reservoirs in wildlife and domestic dogs putting humans and animals at risk. Globally, the overwhelming majority of confirmed human cases are caused by rabies virus (RABV), one of twelve lyssaviruses in the genus *Lyssavirus,* family *Rhabdoviridae*[3] which is transmitted by contact with saliva from infected animals by bite or scratch.

In areas free from terrestrial rabies, it has become extremely rare, with only four imported cases in the UK in the last ten years [4-7].

Once clinical disease develops, rabies is invariably fatal. The Milwaukee Protocol combination of therapies was proposed to aid survival with the exemplar being an unimmunised 15-year old girl with bat rabies in 2004 [8]. The supportive coma-induction, anti-excitatory and antiviral therapies aim to minimise neurological disturbance whilst the immune response confers sterilizing immunity and virus is eliminated. Assessment of the efficacy in subsequent attempts is confounded by variations in stage of presentation, exposure history and treatment regimes [9-15]. Nonetheless, these attempts re-enforce our understanding of rabies pathophysiology, if there are to be further opportunities in building on this initial success in developing an effective treatment protocol. Here, we present a fatal case of human rabies imported into the UK and discuss the rapid diagnosis, virus distribution-associated pathology and our experience with the Milwaukee Protocol.

## Case presentation

A 58-year old woman, of South Asian ethnicity, was referred with painful paraesthesiae in the right forearm, agitation, vomiting, and hydrophobia. During the 4 days prior to admission she attended her general practitioner (GP) and the local emergency department with painful paraesthesiae in her right hand. On the second occasion she was given tramadol; thereafter she began to vomit, refused water, and appeared fearful and agitated. A family member in India suggested that she may have rabies in view of a history of a bite to the right forearm from an ownerless puppy befriended by the patient during a trip to a town near Ludhiana, India, 9 weeks previously. The GP referred the patient to the local emergency department for further management (day 1). The case was discussed with the Health Protection Agency; after an initial blood sample was taken she received vaccine and human rabies immunoglobulin and was transferred to an isolation room in the University College London Hospital intensive care unit.

On arrival she demonstrated pronounced hydrophobia. Although the patient was able to suck on a moist sponge, she gagged and became agitated when water from the sponge dropped onto her bedclothes, or at sight of a cup of water. Initial examination showed dehydration, temperature of 37.9°C, heart rate 120 beats per minute, blood pressure 171/90 mmHg; she was fully conscious. There was no other abnormality on examination. Blood indices showed a mild neutrophilia, urea 7.9 mmol/L, creatinine 79 μmol/L, and normal C-reactive protein. Saliva and skin biopsies from nuchal and bite sites subsequently tested positive for lyssavirus ribonucleic acid (RNA). Over the next few hours the patient developed episodes of extreme agitation despite escalating doses of diazepam (2.5-7.5 mg iv - as frequently as every hour); when one episode became violent she was anaesthetized with standard doses of fentanyl, midazolam, propofol and rocuronium. The majority of elements of the Milwaukee Protocol [15] were commenced. This included drug-induced coma (propofol 200 mg/hr, fentanyl 200 mcg/hr and midazolam 20 mg/hr), neurotransmitter substrate replenishment and antiviral therapy (amantadine 200 mg bd). In addition she received nimodipine 60 mg 4 hourly, sapropoetin 200 mg bd, vitamin C 500 mg and coenzyme Q10 100 mg bd. Continuous electroencephalogram (EEG) monitoring was adopted.

On day 3 she became hypotensive and was commenced on noradrenaline (10-30 mcg/min to maintain a Mean Arterial Pressure (MAP) >80 mmHg). Urine output was responsive, however stroke volume and cardiac output measurements by oesophageal Doppler were not responsive, to fluid boluses. Ketamine sedation (45 mcg/kg/min) was introduced. Adrenaline was added to maintain MAP.

By day 4, passive body warming was required for hypothermia. Ventilatory requirements increased. Escalating doses of ketamine (60 mcg/kg/min), fentanyl (300 mcg/hr) and propofol (70 mg/hr) were required to maintain sedation. Antibiotics were commenced following the suctioning of offensive sputum and evolving radiographic infiltrates.

By day 5, progressive bilateral radiographic infiltrates accompanied worsening hypercapnia. Polyuria with an increasing serum Na^+^ was observed, and treated with oesophageal Doppler-guided fluid optimisation, nasogastric water and intravenous 5% dextrose. Glucose control was required.

On day 6, paralysis (previously avoided due to risk of masking seizures) was achieved with atracurium (10-20 mg/hr) and nitric oxide introduced for intractable hypoxaemia. Escalation of adrenaline (to 80 mcg/min) was required for hypotension, cardiac output started to fall and remained unresponsive. Pupils were dilated and demonstrated sluggish reflex to light. Acidosis and multi-organ failure ensued and haemofiltration was commenced.

On day 8, two episodes of bradycardia required atropine. As noradrenaline increased, hydrocortisone 50 mg qds and fludrocortisone 100 mcg od were started.

On day 9, she developed a junctional rhythm with bradycardic episodes requiring further sedation to ablate the dys-autonomia, atropine boluses, isoprenaline infusion and transvenous pacing.

On day 10, she developed refractory, profound lactic acidosis. Management included 8.4% sodium bicarbonate along with continuous veno-venous haemofiltration. Persistent hypotension was treated with more adrenaline (90 mcg/min) and noradrenaline (160 mcg/min) and addition of argipressin (0.01 u/min). Antimicrobials were escalated and suspected bowel ischaemia was treated supportively as she was unfit for imaging or surgery. The hypotension became refractory and temporary cessation of pacing demonstrated underlying asystole. Supportive treatment was withdrawn.

### Virological findings

#### Antemortem samples

A lyssavirus differential TaqMan© reverse transcriptase(RT)-PCR [16] was used to test for RABV RNA on nucleic acids extracted from tissues. A hemi-nested RT-PCR [17] was used to confirm results and generate product for sequence analysis. The sequence derived was compared to a range of regionally appropriate lyssavirus sequences. Virus was isolated using a standard rabies tissue culture infection test (RTCIT) using neuroblastoma cells as described previously [18]. Viral antigen was detected using the gold standard direct fluorescent antibody test (FAT) with FITC-conjugated antibody (Fujirebio diagnostics) on acetone fixed brain smears, as described previously [18].

Differential TaqMan © RT-PCR [16] demonstrated RABV RNA in all three initial samples (wound biopsy, nuchal skin biopsy and saliva) within six hours of receipt of samples on day 1. Wound biopsy and saliva were strongly positive, with the nuchal biopsy showing lower levels of RNA. A subsequent hemi-nested RT-PCR [17] was negative on nuchal biopsy but positive on wound biopsy and saliva. Subsequent saliva samples taken on day 1 were also positive by rabies TaqMan© and by nested PCR with identical sequences to those derived from initial samples.

**Figure 1:**
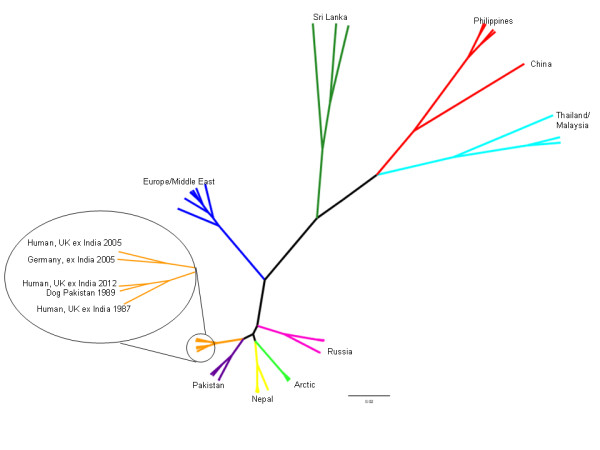
**Phylogenetic relationship between virus isolated in this case (Human ex-India 2012) and a global panel of rabies strains.** A Bayesian Markov–Chain-Monte-Carlo phylogenetic analysis was undertaken using BEAST (v1.8), with the most appropriate tree chosen using Treeanotator and visualized using Figtree. Nodes with posterior probability values over 90% are shown.

Phylogenetic analysis of partial nucleoprotein sequence showed that the virus was a strain in the ‘arctic-like’ lineage 1 and most closely related to sequences derived from dogs in Pakistan in 1979 and 1989, and in another human case imported from India (RV61) (Figure 1).

Virus isolation, attempted on the first saliva sample, was negative. Serum from day 1, before administration of vaccine and immune globulin, was negative tested by the fluorescent antibody virus neutralization test (FAVN) [18] for rabies antibodies, and negative for viral RNA by real-time Taqman PCR (*data not shown*).

#### Autopsy

**Figure 2:**
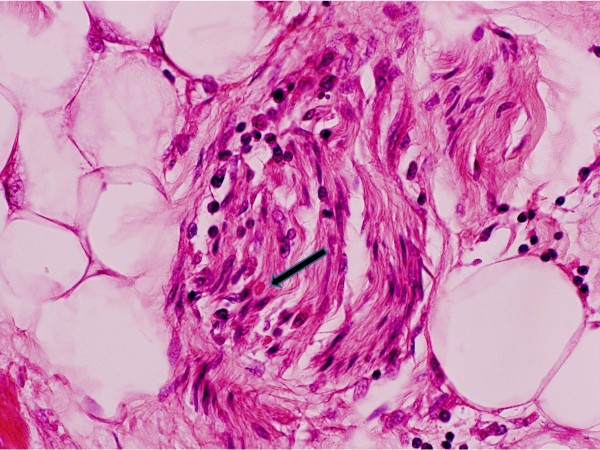
Lymphocytic vagus neuritis in vagus nerve in the heart, with one eosinophilic Negri-like body in the nerve H&E 400×.

**Figure 3:**
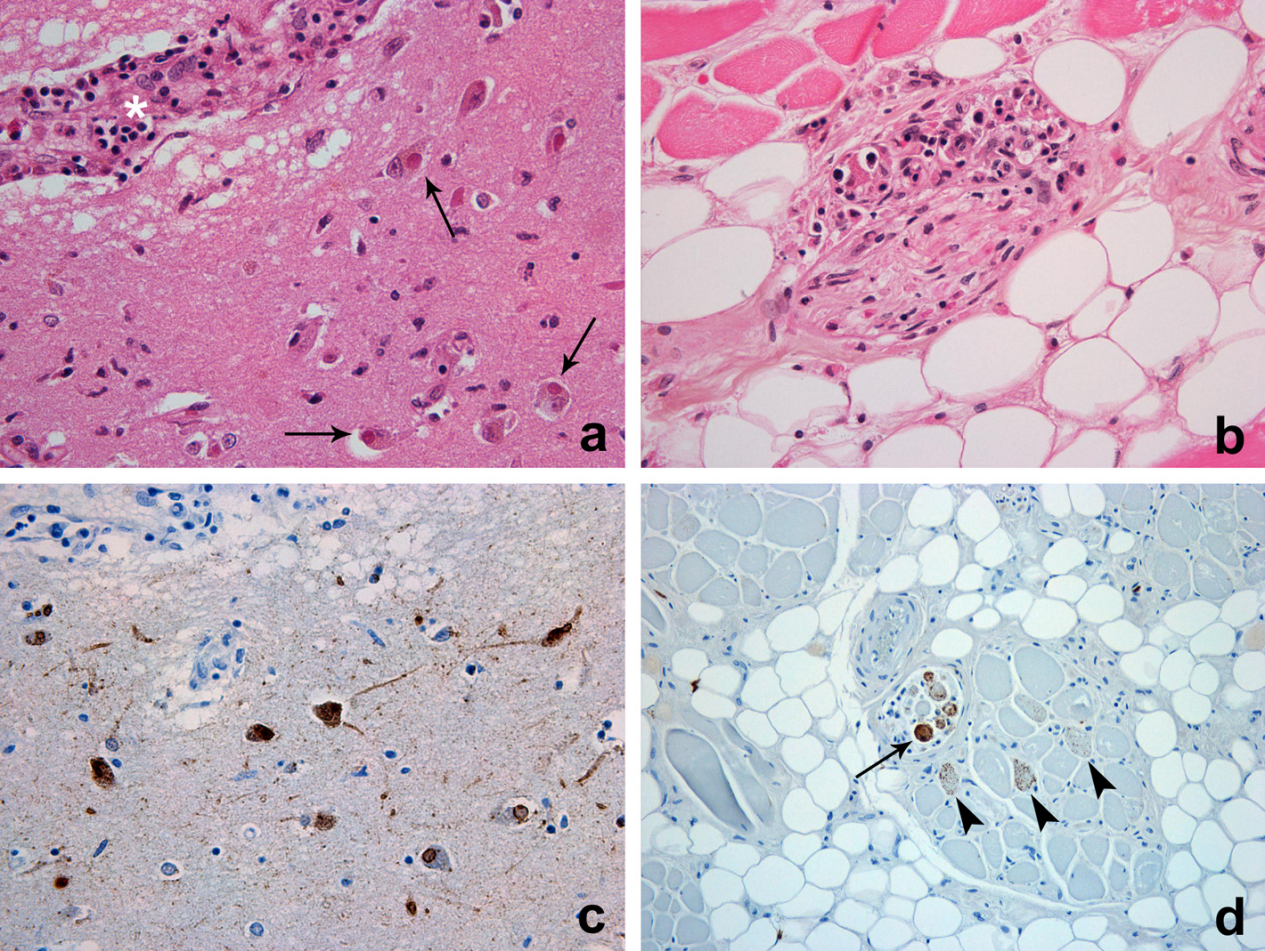
**Post-mortem histology demonstrating distribution of inflammation and rabies virus antigen in brain and muscle. a**. Ganglionitis in autonomic ganglia in the tongue. H&E 400×. **b**. Numerous Negri bodies (arrows) in neurons close to a perivascular cuff in brain tissue (asterisk). H&E 400×. **c**. Immunohistochemical demonstration of rabies antigen in CNS (brown labelling). IHC 400×. **d**. Detection of rabies antigen in muscle spindle (arrow) and skeletal muscle cells in the tongue (arrowheads). IHC 200×.

There was a terminal bronchopneumonia. The heart had mild interstitial T-cell inflammation, and neuritis of vagus nerve branches, including a possible Negri body on Haematoxylin and eosin (H&E) stain (Figure 2), but immunohistochemistry did not confirm this as rabies antigen. The tongue (Figure 3a) and parotid gland also had peri- and endoneural inflammation and ganglionitis.

The brain and meninges were grossly normal; there was no cerebral swelling. Histologically, there was mild encephalitis (T-cell infiltration, perivascular cuffing and microgliosis), most prominent in temporal lobes. Neuronal rabies infection with abundant Negri bodies (round/oval eosinophilic inclusions in the perikaryon) was present in all parts of cerebrum and sub-tentorial brain tissue, most prominently in the temporal lobe and hippocampus (Figure 3b). No hypoxic-ischaemic neurone damage was identified; only rare neurones were undergoing necrosis with sattelitosis.

#### Postmortem samples and rabies identification

RABV antigen and nucleic acid were widely distributed in central nervous system (CNS) and non-CNS tissues post mortem (Figure 3). All brain regions were strongly positive for RABV antigen using FAT; live virus was isolated from brain and cerebrospinal fluid in tissue culture at first passage. No neutralizing antibodies were detected in CSF by FAVN. Although viral RNA was detected in tongue, parotid salivary gland and a mouth swab taken post mortem, live virus was not recovered from these samples. Viral RNA was not detected in heart tissue.

Immunohistochemical demonstration of RABV antigen in post-mortem samples from hippocampus, cerebellum, medulla, tongue, parotid gland and myocardium was performed as described previously [19]. Profuse antigen was observed in the perikaryon and neuropil in all CNS regions, including all cranial nerve roots identified (Figure 3c). Abundant antigen was observed in autonomic ganglia, muscle spindles and skeletal muscle fibres in the tongue (Figure 3d). Limited amount of immunolabelling was observed in nerve axons in the parotid gland. No labelling was observed in lingual epithelium or parotid acinary cells (*data not shown*).

## Conclusion

This is the fifth case of rabies in the UK since 2000 and highlights the need for better education of travellers and clinicians on the risks of travel-related acquisition.

Rabies is a vaccine-preventable disease. UK imported rabies cases, acquired through animal contact in rabies endemic countries, have been uniformly fatal and were noted to have not received pre- or post-exposure prophylaxis (PEP) [20]. Financial cost has been cited as a reason for declining pre-exposure immunization [21] and therefore, when appropriate, the advantages need to be clearly communicated to justify the cost to the patient. The basis for poor post-exposure prophylaxis remains unclear: a recent Geosentinel survey of 23,509 travellers found that 320 travellers sustained animal-related injuries (mainly in Asia involving dogs) yet only 66% received PEP [22].

This case illustrates the insidious and nonspecific symptoms that may precede rabies and reminds clinicians to consider the diagnosis. Lack of awareness of potential rabies exposure compounded with the nonspecific prodrome that may precede neurological signs mean that delayed clinical suspicion is frequent [23].

Phylogenetic analysis demonstrated that the virus isolated from this patient was from a canine lineage of viruses thought to have evolved 500 years ago from strains in Polar regions, and now a dominant strain in parts of Asia [24,25]. The virus isolated here is very similar to previous imported cases from India ten years previously [6] and similar to cases in dogs in 1970s-1980s (Figure 1). This implies that current strains are very similar to those circulating in stable endemic independent cycles in the region for the past 30 years. An average of only 57 rabies cases have been reported in animals from the whole of India annually since 2005 [26], and there is therefore likely to be significant underreporting. This case illustrates the growing divide between countries where rabies is endemic and underreported, and those where rabies is extremely rare.

Until 2005, some form of prophylaxis was documented in the handful of patients who survived clinical rabies [27-31]. However, in 2004, in the absence of vaccination, a 15-year-old girl with rabies, diagnosed on the basis of a history of bat bite and anti-rabies antibodies in the CSF, survived after the use of the Milwaukee Protocol [8]. The protocol comprised therapeutic bundles based on rebalancing an apparent rabies-induced tetrahyrobiopterin deficiency that leads to dopamine and serotonin deficiency and poor nitric oxidase activity [32] whilst the natural immune response clears the virus, as reported in animal models showing immune-mediated viral clearance from the central nervous system and T-cell mediated neuronal apoptosis [33-37].

The logistical complexities of adoption of the Milwaukee Protocol are substantial, and this report provides additional data on its lack of efficacy. Despite revisions to reduce adverse reactions to component drugs [13,32,38], patients treated by the Protocol and submitted on a central database [39] number 43, of which only five (excluding the first survivor) have been registered as survivors. Multiple failed attempts have been described [9,11-13,40-42] including the last UK imported case [7] but the potential for efficacy has been debated due to case by case protocol deviations. In the absence of animal trial data, the only evidence for the protocol has emerged from individual case review. Specific factors that appear relevant include: therapy with rabies vaccine prior to onset of symptoms, young age, lack of comorbidity, infection with bat rabies variant, early evolution of neutralizing antibodies in serum and CSF, and mild neurological disease [8,13,43-45]. Also relevant is the immune response, as asymptomatic seroconversion and abortive infection have been described [46-48]. In our case, the above positive prognostic factors were absent. Furthermore, our case was treated with vaccine and immune globulin prior to institution of the recommended protocol, which may have resulted in delay in generation of the host immune response. As in other cases, it was recommended initially (prior to disease confirmation) as part of the standardized post-exposure protocol. This patient had no serum antibody on presentation, and failed to develop detectable antibody levels in CSF by day 8, which supports previous evidence that lack of CSF antibody is a poor prognostic indicator. It is unknown whether the patient would have developed an effective immune response given sufficient time. We acknowledge that, in future suspected cases, post exposure prophylaxis should await results of diagnostic tests to give the protocol optimum chance.

The actual mode of this patient’s death appears to have been profound dysautonomia. Despite anticipating it, we were ultimately unable to control it. Perhaps an earlier and even more aggressive approach to dampen this autonomic dysfunction may have changed the outcome, but elements of the Milwaukee Protocol are at odds with this therapeutic strategy. Other elements were just not feasible on our ICU. Adoption of the Protocol remains contentious. It may be that individualization of care in future cases is possible with the identification of prognostic biomarkers, and recent metabolomic studies on the spinal fluid of 2 survivors versus 7 non-survivors have proposed several biomarkers [49].

Viral distribution studies in animal models, and through quantitative PCR in this case, demonstrate that virus can be widely distributed. There was no detectable RNA in the heart muscle, albeit from only one sampled region (*data not shown*), however. virus replication centres (Negri bodies) in the cardiac vagus nerve were detected histologically (Figure 3). Previously described autopsy studies of cardiac tissue have shown the presence of rabies viral antigen deposits in the ganglia of three cases, with concurrent ganglioneuritis and myocarditis seen in two and one of the cases respectively - from which it was inferred that centrifugal neuronal spread of the rabies virus occurred with subsequent spread to the myocardium [50]. Similarly, clinical and autopsy data in this case support the spread of virus but do not conclusively prove presence of virus in the heart. The anterograde axonal spread of virus is not well understood: it is recognised that the virus will spread via the autonomic nervous system to a wide range of organs including the heart [51]; and that the latter is typical for the clinical course of rabies in humans and leads to myocardial dysfunction and death.

In this case, the autopsy demonstrated the previously documented conundrum that death with brain involvement occurs despite minimal encephalitis and neuronal >loss [52]. However, the marked brainstem involvement recently described in studies of viral antigen distribution and magnetic resonance imaging (MRI) in rabies patients [53-55] and implicated as potentially relevant to centrally-mediated dysautonomia was not seen here.

Consensus regarding the Milwaukee Protocol in patients with canine rabies will evolve as more outcomes are published. In the interim, the emphasis of management is based on timely diagnosis to optimise supportive care. The prompt availability of the sequence and phylogenetic analysis of the virus also aided clarification of the source as this was not initially clear. Much can be learnt from the promptness of confirmation of diagnosis in this case, as well as the efficient and daily coordination of information between specialist and referring hospitals, public health authorities and national experts which was crucial in this patient’s care and minimization of onward transmission. This highlights the benefit of discussion on a daily basis by relevant experts and fully encompasses the ‘One Health’ agenda [56] for rare imported human infectious diseases.

## Consent

Written informed consent was obtained from the patient’s next of kin for publication of this Case Report and any accompanying images. A copy of the written consent is available for review by the Editor-in-Chief of this journal.

## Abbreviations

RNA: Ribonucleic acid; CSF: Cerebrospinal fluid; PCR: Polymerase chain reaction; RABV: Rabies virus; GP: General practitioner; EEG: Electroencephalogram; MAP: Mean arterial pressure; RT-PCR: Reverse transcriptase polymerase chain reaction; RTCIT: Rabies tissue culture infection test; FAT: Fluorescent antibody test; FITC: Flourescein isothyanate; PEP: Post-exposure prophylaxis; H&E: Haematoxylin and eosin; CNS: Central nervous system; FAVN: Fluorescent antibody virus neutralization test.

## Competing interests

All authors declare that they have no competing interests.

## Authors’ contributions

SmP, DH, MB and ARF drafted the manuscript. DH, AN, EW & ARF carried out the molecular virology studies, participated in the sequence alignment and drafted the virological sections of the manuscript. MB, EN, SQ, SmP, SaP, DH, ARF, DW and DB researched the literature, co-ordinated the management of the case, discussed the scientific issues around management and helped to draft the manuscript. All authors read and approved the final manuscript.
